# Perspectives from patients with chronic lung disease on a telehealth-facilitated integrated palliative care model: a qualitative content analysis study

**DOI:** 10.1186/s12904-024-01433-3

**Published:** 2024-04-18

**Authors:** Jeannette Kates, Carrie Tompkins Stricker, Kristin L. Rising, Alexzandra T. Gentsch, Ellen Solomon, Victoria Powers, Venise J. Salcedo, Brooke Worster

**Affiliations:** 1 College of Nursing, Thomas Jefferson University, 901 Walnut Street, Suite 702, Philadelphia, PA 19107 USA; 2 Canopy Cancer Collective, P.O. Box 3141, Saratoga, CA 95070 USA; 3 Center for Connected Care, Thomas Jefferson University, 1025 Walnut Street, Suite 300, Philadelphia, PA 19107 USA; 4 Department of Emergency Medicine, Sidney Kimmel Medical College, Thomas Jefferson University, 132 South 10th Street, Philadelphia, PA 19107 USA; 5 Department of Internal Medicine, Hospital of the University of Pennsylvania, 3400 Spruce Street, Philadelphia, PA 19104 USA; 6 Department of Medical Oncology, Sidney Kimmel Cancer Center, Thomas Jefferson University, 925 Chestnut Street, Suite 420A, Philadelphia, PA USA

**Keywords:** Palliative care, Chronic lung disease, Telehealth

## Abstract

**Background:**

Chronic lung disease affects nearly 37 million Americans and often results in significant quality of life impairment and healthcare burden. Despite guidelines calling for palliative care (PC) integration into pulmonary care as a vital part of chronic lung disease management, existing PC models have limited access and lack scalability. Use of telehealth to provide PC offers a potential solution to these barriers. This study explored perceptions of patients with chronic lung disease regarding a telehealth integrated palliative care (TIPC) model, with plans to use findings to inform development of an intervention protocol for future testing.

**Methods:**

For this qualitative study, we conducted semi-structured interviews between June 2021- December 2021 with patients with advanced chronic lung disease. Interviews explored experiences with chronic lung disease, understanding of PC, and perceived acceptability of the proposed model along with anticipated facilitators and barriers of the TIPC model. We analyzed findings with a content analysis approach.

**Results:**

We completed 20 interviews, with two that included both a patient and caregiver together due to patient preference. Perceptions were primarily related to three categories: burden of chronic lung disease, pre-conceived understanding of PC, and perspective on the proposed TIPC model. Analysis revealed a high level of disease burden related to chronic lung disease and its impact on day-to-day functioning. Although PC was not well understood, the TIPC model using a shared care planning approach via telehealth was seen by most as an acceptable addition to their chronic lung disease care.

**Conclusions:**

These findings emphasize the need for a patient-centered, shared care planning approach in chronic lung disease. The TIPC model may be one option that may be acceptable to individuals with chronic lung disease. Future work includes using findings to refine our TIPC model and conducting pilot testing to assess acceptability and utility of the model.

**Supplementary Information:**

The online version contains supplementary material available at 10.1186/s12904-024-01433-3.

## Introduction

Nearly 37 million people in the US live with chronic lung disease, and patients with chronic lung disease experience healthcare burdens that escalate in the last months and years of life [1–3]. Chronic lung diseases affect the airways and other structures of the lungs [4]. Some of the most common chronic lung diseases are asthma, chronic obstructive pulmonary disease (COPD), and occupational lung diseases. Although they are not curable, various treatments are available to improve disease-related symptoms. There is an under-recognized need for palliative care (PC) for people with chronic lung disease, who may experience weight loss, exercise intolerance, impaired quality of life, and increased health-related costs [5]. The inclusion of PC and advance care planning in people with advanced illness has consistently demonstrated alleviation of many healthcare burdens, improvement of health-related quality of life, and decreased costs associated with serious illness [6–10]. Although large, multi-site randomized controlled clinical trials of PC in chronic lung disease are a gap in the literature, there are some smaller studies that have demonstrated fewer hospital deaths, reduced costs, and increased mastery of breathlessness in COPD [5]. As such, guidelines and recent reports have called for PC integration into pulmonary care before the end of life, including for symptom relief [11].

Although PC is important for patients with chronic lung disease, studies have found that less than 2% of those individuals receive specialized PC during their illness trajectory [12]. There is a range of identified barriers to effectively integrating PC into specialty pulmonary care. The existing PC workforce has limited capacity, and thus cannot reasonably meet the needs of the growing populations with chronic lung disease (13). Moreover, existing models of outpatient PC are insufficient to meet the needs because they can add burden on to seriously ill patients and their families by requiring travel for additional in person appointments and because they often lack connectivity to primary care or specialty physicians [14, 15]. Although alternative models, such as integrated palliative home care and pulmonary specialist-provided primary PC, are beginning to be developed and tested to meet the needs of this large and high-morbidity chronic lung disease population [16, 17], none addresses the workforce constraints.

Thus, there is a critical need for innovative and scalable solutions to integrate PC into standard pulmonary disease management with a patient-centered focus. Multi-level interventions that incorporate telehealth and use of PC specialty services are best poised for scale and sustainability, particularly if developed with the intent of building capacity for PC knowledge and skills within the primary medical home. To that end, our team developed a telehealth-facilitated integrated PC model (TIPC) that is designed to deliver needed PC services to patients with chronic lung disease in an approach that is integrated with the patients’ primary pulmonary providers while also addressing limited PC specialty bandwidth. The objective of this study was to elicit patient feedback on the proposed TIPC model to ensure patient-centeredness of the model before implementation in a future trial. This qualitative interview study explored the perceived acceptability and anticipated facilitators and barriers of the proposed TIPC model.

## Methods

This study took place in a large academic medical center in Philadelphia, PA after approval by Thomas Jefferson University Institutional Review Board. Patients were included if they were English-speaking, diagnosed with non-cancerous chronic lung disease (e.g., COPD/emphysema, asthma, and interstitial lung disease), and had been hospitalized within the past six months at a hospital within the health system where this study took place or were identified by their provider as having advanced disease that would potentially benefit from a PC consult. Clinicians from the Department of Medicine, including the Division of Pulmonary, Allergy and Critical Care Medicine, Division of Hospital Medicine, and Division of Internal Medicine, identified patients for recruitment. Recommended patients were screened via their electronic health record (EHR) for eligibility, and potentially eligible patients were contacted via telephone and follow-up calls conducted every three to seven days for up to three contact attempts. During the recruitment phone call, a research team member assessed each patient’s interest and confirmed study eligibility. Participants’ caregivers were also invited to participate if available and if the patient wanted them present. Verbal consent was obtained using teach-back questions to ensure full understanding. We developed a semi-structured interview guide to assess experience with chronic lung disease, knowledge and attitudes about PC, and feedback about the proposed TIPC model (See Appendix). The guide was drafted and refined by the entire research team applying their clinical expertise (JK, CTS, KLR, BW) as well as prior literature. The guide was tested informally among the team, though was not formally pilot tested with patients. Basic demographic data were also collected. Patient participants received $25 for study participation. Recruitment continued until thematic saturation was reached. None of the research team members had a pre-existing relationship with any of the participants.

### Telehealth-facilitated integrated palliative care model (TIPC)

Our proposed TIPC model was explained to participants during the semi-structured interviews. The verbiage used to describe the model to participants is included in the interview guide (See Appendix). The TIPC model comprises two telehealth-facilitated PC visits that directly engage the patient, family, and pulmonologist in patient-centered care planning. The initial telehealth visit takes place between the patient and/or their caregiver and a specialty PC provider. During this visit, the patient talks about their illness and needs related to their illness, with the visit guided by the Serious Illness Conversation Guide [18] This may include exploring their goals, preferences for care, fears and worries, bothersome symptoms or limitations, and family involvement. A summary of the visit is compiled by the PC clinician, detailing the patient’s most salient preferences or goals identified in this visit as well as a proposed plan for meeting these goals and/or other concerns. These findings are then communicated to the patient’s primary pulmonary team via electronic communication in the EHR. Thereafter, a collaborative three-way visit is scheduled to include the patient and/or caregiver, specialty PC provider, and one or more of the patient’s pulmonary team members. The goals of this visit are to ensure communication of the patient’s preferences directly to the pulmonary care team, enabling the pulmonary team to gain a better understanding of any needed changes to the patient’s treatment plan to facilitate better meeting the patients’ goals. This approach can be immensely beneficial to patients and their family by facilitating clear communication at a time when the patients are not critically ill and hospitalized, which is often far too late in the course of an illness to have useful conversations.

### Data analysis

Interviews were audio-recorded, professionally transcribed, and reviewed by a research team member for accuracy. Transcripts were then uploaded into qualitative data analysis software, NVivo [19]. The research team worked together to develop a preliminary codebook based on the first two interviews, and systematically refined it through an iterative process until a final codebook was established. The research team included expertise in qualitative research (KLR, AG), palliative care providers (JK, CTS, BW), and telehealth providers (JK, KLR, BW). Interviews were analyzed with an inductive approach by two trained coders using conventional qualitative content analysis [20]. Transcripts were double-coded to ensure uniform codebook application between coders and inter-coder reliability was confirmed with the kappa coefficient (κ). A mean κ value of 0.61 to 0.80 is considered substantial agreement and this range was used as a guide to assess sufficient inter-coder agreement. The research team met regularly for debriefing, and any discrepancies were resolved through team consensus. An audit trail of coding decisions was maintained by the study coordinator. Member checking of findings with participants was not performed. Demographic characteristics were summarized with descriptive statistics.

## Results

We attempted to contact 48 identified patients, 35 of whom were successfully contacted. Ultimately, 21 patients agreed to participate, and 20 interviews were analyzed as it was later determined that one interviewed participant was ineligible due to a cancer diagnosis. Approached patients were excluded if they had a non-functioning phone number (*n* = 3), were unable to be reached after three attempts (*n* = 10), were unable to speak on the phone (*n* = 1), were no longer a patient of the health system at which recruitment was being conducted (*n* = 1), were found to be ineligible at time of contact due to active cancer treatment (*n* = 3), were currently receiving hospice or outpatient palliative care (*n* = 2), reported no diagnosis of lung disease (*n* = 2), or declined participation (*n* = 6). The average length of interviews was 31 min. 40% of transcripts were double-coded and the average κ for double coded interviews was 0.94. Participant demographics are included in Table 1.

**Table 1:** Patient demographics (*n* = 20)

Characteristic	n (%)
**Age - Mean (SD)**	64 (10.9)
**Gender Identity** Female Male	14 (70%) 6 (30%)
**Race**	
Black	9 (45%)
White	10 (55%)
**Ethnicity** Hispanic or Latino	1 (5%)
**Years with chronic lung disease - Mean (SD)**	7.5 (6.4)
**Chronic lung disease diagnosis** Chronic obstructive pulmonary disease (COPD)/emphysema only Interstitial Lung Disease (ILD) only COPD/emphysema + Asthma COPD/emphysema + ILD	10 (50%) 6 (30%) 3 (15%) 1 (5%)
**Hospitalized within last 6 months**	15 (75%)
**Income** <$10,000 $10,000-$24,000 $25,000-$49,000 $50,000-$99,000 Decline	5 (25%) 4 (20%) 6 (30%) 1 (5%) 4 (20%)
**Number of people in household- Mean (SD)**	2.7 (1.3)
**Education**	
Less than High School	3 (15%)
High School	14 (70%)
College	3 (15%)
**Employment status**	
Looking for work	2 (10%)
Retired	9 (45%)
Disabled	9 (45%)
**Self-Rated Physical Health- Mean (SD)** Likert Scale 1 (poor) − 5 (excellent)	2.2 (0.8)
**Self-Rated Mental Health- Mean (SD)** Likert Scale 1 (poor) − 5 (excellent)	2.9 (1.0)

Qualitative results are presented in three categories: burden of chronic lung disease, understanding of PC, and perceived acceptability of PC and TIPC intervention. Table 2 lists categories and representative quotes.

**Table 2:** Representative quotes for identified categories

Category		Participant quote
Burden of chronic lung disease	Dyspnea	“I just wish there was something that I could not have this problem with trying to walk and can’t breathe. Because I need to be able to get up and go play with my plants, dust my tables off myself. Little things that I would like to do and it’s just not able to do.” [Participant 17]
	Medication adverse effects	“And honestly…I thought to myself I’d rather be dead than being on this drug. It was that bad. It wasn’t living.” [Participant 6]
	Oxygen therapy management	“I’ve been in the house 99.9% of the time; I haven’t gone out. Because these other tanks are bulky, they’re hard to maneuver and they only last like an hour, not even.” [Participant 8]
	Social isolation	“You’re eliminated from a lot of things. If you went to a concert and you had to get up and go to the bathroom, you wouldn’t be able to do it.” [Participant 17] “I worry my not breathing right is affecting her.” [Participant 4]
	Grief, sadness, anxiety	“Sometimes I just want to take a couple pills and go to sleep. You know what I mean? But I don’t because of my granddaughters. If it wasn’t for them, I probably would’ve by now.” [Participant 4] “I can’t catch breath at all…It’s scary, believe me.” [Participant 5]
	Challenges with care	“I’m so confused. I don’t know who to listen to now.” [Participant 16] “The one doctor would say, well, I want to try this, this and this. And then another doctor will say, well, I also want to try this, this and this. But if it don’t work, or if it does work, then you either stay with it or if it don’t work, then the plans have got to change. That’s something that I believe – I actually think that it gets lost between the doctors.” [Participant 12] “Not everyone is like my father at 86 years old… He has the drive to get better or at least stay stable and live his life to the fullest… I think they’re treated or passed over because they are older…” [Participant 14]
Understanding of palliative care		“Palliative care is nursing care to me…It’s all palliative care, as far as I’m concerned” (referring to nursing care in the hospital) [Participant 3] “They would walk you around…checking oxygen” [Participant 15]
Perceived acceptability of palliative care and the TIPC intervention	Openness to palliative care	“It would be nice to be able to kind of know where my future kind of may go, doesn’t necessarily mean it’s going to go there. But it could be something to think about, that I might want to do something now to get ready for” [Participant 10] “At this point I’m willing to try anything to help. Okay? This is how miserable I am.” [Participant 16] “Sometimes I think going three months without seeing him [physician] is a long time because I’m not sure what my lungs are – like, the timing of my lungs. You know what I’m saying? As far as, like, how long do I have before they stop working. Those types of questions… I think if I had somebody in the middle that can help answer those and we can figure those out together, that my worries will kinda calm down.” [Participant 1]
	Advantages to telehealth	“Going out…It can be a real chore. So I think having this on video, phone, I think it’s a lot better than having to do it in person….” [Participant 6] “I would love to have video calls and things, especially with my daughter being involved with it.” [Participant 11]
	Concerns about telehealth	“I just don’t feel comfortable about being videoed.” [Participant 7] “It [in-person visit] seems more personal to me” [Participant 19]
	Shared care planning	“You have to come together. We should be as one. All of us should be on the same page… You would want everybody to work together to be as one.” [Participant 16] “It’s really smart. That’s the way it should be….They would…probably tell the doctor. This guy needs a little more uplifting…Let’s try to make him feel a little bit better because…negative isn’t working with him, but positive is. You know, I think that’s really important.” [Participant 8]

### Burden of chronic lung disease

Patients discussed the impact that living with chronic lung disease has on their everyday lives (Table 2). 90% of patients discussed the physical limitations they experience due to living with chronic lung disease, of which most patients (*n* = 16) specifically discussed the difficulty of living with dyspnea. Patients reported limitations in activities of daily living such as toileting and instrumental activities of daily living such as doing laundry. Additionally, patients described the limitations dyspnea put on participating in hobbies like fishing or gardening.

In addition to the limitations of dyspnea, patients discussed the limitations imposed by their medications and/or oxygen therapy. Patients mentioned feeling confined to their homes due to oxygen therapy and experiencing reduced quality of life due to medication adverse effects. Several patients described how they have fewer social opportunities due to their symptoms and/or treatment, while one patient expressed concern about whether her inability to play with her granddaughter is affecting her granddaughter. When talking about their experiences living with chronic lung disease, five patients expressed feelings of grief and sadness about not being able to do what they used to do and the subsequent loss of their independence.

More than half of patients identified challenges with care, including availability of treatment options, lack of communication with providers, and lack of coordination between providers. One patient and his daughter mentioned experiencing ageism in his care, while another patient expressed the importance of instilling hope in new trials or medications instead of being “all negative.” Several patients expressed frustration about lack of communication and coordination between doctors involved in their care, such as apparent contradictions in medications between the inpatient and outpatient setting as well as between the primary care provider and the outpatient pulmonologist.

### Understanding of PC

All patients were asked if they had ever heard of PC prior to the interview. 90% of patients had never heard the term “palliative care.” The two patients who were familiar with the term demonstrated a misunderstanding of the term, with one describing it as simply “nursing care” and the other as being walked around to have your oxygen checked (Table 2). After providing a definition of PC from the widely accepted terminology developed by the Center to Advance Palliative Care [21] (see Appendix), most patients continued to demonstrate confusion regarding the role of PC.

### Perceived acceptability of PC and of TIPC intervention

85% of patients were open to participating in the proposed intervention once it was explained (Appendix). Patients expressed interest in the increased access to PC provided by the TIPC intervention. Telehealth as a delivery platform was seen as both a facilitator and a barrier. From the facilitator perspective, a majority (55%) of participants reported using telehealth in some capacity to communicate with their care team. In the context of the TIPC intervention, almost all patients would feel comfortable doing the visit over telehealth, and several advocated in favor of the virtual format (Table 2). Those in favor welcomed the convenience of not having to attend an in-person visit and being able to have family members attend virtually. Patients who reported use of telehealth in the past did not find the technology difficult to use upon getting used to it. Other factors promoting acceptability of the TIPC model included patients wanting additional support with disease-related symptoms, conversations about disease trajectory, and facilitated communication with pulmonary specialists.

From the barrier perspective, the few participants who were opposed to telehealth voiced concern about ease of use, video transmission, and lack of personal touch. The other primary barrier to potential TIPC model uptake was an overall lack of understanding of PC. Individuals who were opposed to participating in the intervention did not perceive its utility in light of their current circumstances and either stated they simply did not need it, or they could not see the benefits.

## Discussion

In this qualitative study, we explored the perceptions of 20 patients with chronic lung disease about their potential use of a TIPC model as an adjunct to their existing care for chronic lung disease. Participants overall reported a high disease burden related to their disease, with many describing impacts on their physical and psychosocial functioning. While we found that, in general, participants had a limited understanding of PC, most participants were interested in the TIPC model upon explanation. Facilitators of uptake of our proposed TIPC model included the desire for additional support for symptom management, conversations about disease trajectory, and facilitated communication with pulmonary providers. Lack of understanding of PC was the primary overall barrier to uptake. Telehealth as the platform for the intervention was both a facilitator and a barrier.

Literature has demonstrated the association between symptoms of chronic lung disease and clinically meaningful decline in quality of life, overall health status, and prognosis of those with chronic lung disease [22]. Data from a multicenter, prospective study of patients with COPD showed that significant increases in COPD respiratory symptoms (dyspnea, coughing, and expectoration) were associated with deterioration of health-related quality of life [23]. Additionally, patients with chronic lung disease experience worse psychological functioning and greater psychological distress than patients with other chronic medical conditions [24]. Although quality of life was not explicitly explored in our study, patients reported similar impacts of chronic lung disease on their physical, social, and emotional wellbeing. Despite the potential benefits of PC on symptom burden in this population, patients with COPD rarely receive it. One of the clinical barriers to PC referral from pulmonary specialists is a misplaced fear that PC clinicians will overprescribe medications that could lead to respiratory suppression in patients with chronic lung disease [14, 25]. Our shared-care approach in the TIPC model may help allay those fears, while elucidating patients’ goals related to symptom management.

Our study findings suggest that patients with chronic lung disease are receptive to conversations about their goals and preferences and appreciate the shared care planning approach in our proposed TIPC model. Best practice for communication about serious illness involves exploring illness understanding, eliciting decision-making preferences, understanding patients’ priorities and goals, exploring views on trade-offs and wishes for family-caregiver involvement, and sharing information about prognosis in line with their preferences [26, 27]. Awareness of individual communication preferences is crucial because not all patients express openness to end-of-life issues [28]. In qualitative studies [29, 30], patients with chronic lung disease expressed concerns about not receiving education about disease progression and end of life, which can prompt anxiety about what the end of life will entail. When end-of-life discussions do occur, the quality of communication is rated highly, suggesting the key to improving communication is to overcome initial barriers that prevent these discussions from taking place [30]. There has been a call for collaborative, integrated approaches to PC in chronic lung disease care [28], such as we propose in the TIPC model.

In our study, the use of telehealth as the delivery method for PC was both a facilitator and a barrier. Despite a significant uptake in telehealth over the last several years, numerous barriers persist. Although digital access and digital literacy are important disparities to consider, these were not the only concerns addressed by patients in our study. Our findings also showed concerns related to not wanting to be videotaped and lack of personal connection, which was in line with prior research demonstrating that trust and cultural factors affect telehealth uptake [31]. Thus, telehealth and the TIPC intervention as proposed will not be acceptable to all patients with chronic lung disease. One adaptation that we could consider is offering a telephonic option as an alternative to video. Iyer and colleagues [33] have demonstrated acceptability and feasibility of a telephonic nurse-led early palliative care intervention for COPD.

It is important to note that lack of understanding of PC by patients was a barrier to our proposed TIPC model and is thus likely a barrier to uptake of PC in general. In our study, only 10% (*n* = 2) of patients were familiar with PC, and both inaccurately described it. Research has shown that less than 30% of adults nationally report knowing about palliative care, and only 12.6% report knowing what palliative is and hold no misconception [32]. Misconceptions by pulmonary clinicians have also been reported in the literature [14]. Thus, strategies aimed at increasing awareness and countering broader public misconceptions must include efforts to also address clinician misconceptions [32].

This study adds to an emerging body of literature about the use of telehealth for PC in chronic lung disease. Consistent with other studies [33, 34], our study found that telehealth was an acceptable way in which to facilitate a PC visit with patients with chronic lung disease among most patients. Most participants in our study highlighted the convenience of telehealth, especially considering the symptom burden of chronic lung disease and difficulties with leaving the house.

Limitations.

This study has limitations. Participants were recruited from a single urban academic medical center and results may not be generalizable to other populations and settings, particularly those in rural and nonacademic settings. Future work is needed to test this model across a broader range of settings. Additionally, since the interviewers were from the same institution from which patients receive their care, there may be a social desirability bias. Another limitation is that the interview guide was not pilot tested with patients. Furthermore, although results from this study will inform the refining of the TIPC model, patient and caregiver input was not sought for the model presented to participants in this study. Finally, 75% of enrolled participants had been hospitalized within the past six months, potentially biasing responses to be more favorable towards PC than among a group of patients who have not required recent hospital-level care.

## Conclusion

In conclusion, this study explored the perceived acceptability and anticipated facilitators and barriers of our proposed TIPC model in patients with chronic lung disease. Analysis revealed the impact of chronic lung disease on day-to-day functioning and a gap in understanding of PC, with our proposed TIPC model thought to be an acceptable addition to chronic lung disease care for most of the participants. These findings emphasize the need for a patient-centered, shared care planning approach in chronic lung disease. This work will inform the next phase of our work in which we test implementation of the TIPC model in patients with chronic lung disease.

### Electronic supplementary material

Below is the link to the electronic supplementary material.

Supplementary Material 1

## Data Availability

The datasets generated and analyzed during the current study are available from the corresponding author on reasonable request.

## Abbreviations

PC: Palliative Care
TIPC: Telehealth-facilitated Integrated Palliative Care
COPD: Chronic Obstructive Pulmonary Disease
EHR: Electronic Health Record

## References

[1] American Lung Association Impact. | American Lung Association [Internet]. [cited 2023 Feb 9]. https://www.lung.org/about-us/our-impact.

[2] Iyer AS, Goodrich CA, Dransfield MT, Alam SS, Brown CJ, Landefeld CS (2020). End-of-life spending and Healthcare utilization among older adults with chronic obstructive Pulmonary Disease. Am J Med.

[3] Baltaji S, Cheronis N, Bajwa O, Cheema T (2021). The role of palliative care in COPD. Crit Care Nurs Q.

[4] WHO Chronic respiratory diseases. https://www.who.int/health-topics/chronic-respiratory-diseases#tab=tab_1.

[5] Iyer AS, Sullivan DR, Lindell KO, Reinke LF (2022). The role of Palliative Care in COPD. Chest.

[6] Dionne-Odom JN, Ejem DB, Wells R (2020). Effects of a telehealth early palliative care intervention for family caregivers of persons with advanced heart failure: the ENABLE CHF-PC randomized clinical trial. JAMA Netw Open.

[7] Beasley A, Bakitas MA, Edwards R, Kavalieratos D (2019). Models of non-hospice palliative care: a review. Ann Palliat Med.

[8] Bakitas MA, Dionne-Odom JN, Ejem DB (2020). Effect of an early palliative care telehealth intervention vs usual care on patients with heart failure: the ENABLE CHF-PC randomized clinical trial. JAMA Intern Med.

[9] Quinn KL, Shurrab M, Gitau K, Kavalieratos D, Isenberg SR, Stall NM (2020). Association of Receipt of Palliative Care Interventions with Health Care Use, Quality of Life, and Symptom Burden among adults with chronic Noncancer illness: a systematic review and Meta-analysis. JAMA.

[10] Masqood MH, Khan MS, Warraich HJ (2021). Association of palliative care intervention with health care use, symptom burden and advance care planning in adults with heart failure and other noncancer chronic illness. J Pain Symptom Manage.

[11] de Oliveira EP, Medeiros P. Palliative care in pulmonary medicine. J Bras Pneumol., Hertz P, Bond A, McDermid RC, Celi LA. Use of Palliative Care in Patients With End-Stage COPD and Receiving Home Oxygen: National Trends and Barriers to Care in the United States. Chest. 2017;151(1):41–6. 10.1016/j.chest.2016.06.023 PMC6026227 27387892

[12] Rush B, Hertz P, Bond A, McDermid RC, Celi LA. Use of Palliative Care in Patients With End-Stage COPD and Receiving Home Oxygen: National Trends and Barriers to Care in the United States. Chest. 2017 Jan;151(1):41–6. 10.1016/j.chest.2016.06.023 PMC6026227 27387892

[13] GeriPal. Proactive Integration of Geriatrics & Palliative Care Principles into COPD: Podcast with Anand Iyer on Apple Podcasts [Internet]. [cited 2023 Feb 9]. https://podcasts.apple.com/us/podcast/proactive-integration-geriatrics-palliative-care-principles/id1164272877?i=1000473180017.

[14] Etkind SN, Bone AE, Gomes B, Lovell N, Evans CJ, Higginson IJ (2017). How many people will need palliative care in 2040? Past trends, future projections and implications for services. BMC Med.

[15] Iyer AS, Dionne-Odom JN, Khateeb DM, O’Hare L, Tucker RO, Brown CJ (2020). A qualitative study of pulmonary and palliative care clinician perspectives on early palliative care in chronic obstructive pulmonary disease. J Palliat Med.

[16] Kavalieratos D, Georgiopoulos AM, Dhingra L, Basile MJ, Rabinowitz E, Hempstead SE (2021). Models of Palliative Care Delivery for individuals with cystic fibrosis: Cystic Fibrosis Foundation evidence-informed Consensus guidelines. J Palliat Med.

[17] Scheerens C, Pype P, Van Cauwenberg J, Vanbutsele G, Eecloo K, Derom E (2020). Early Integrated Palliative Home Care and Standard Care for End-Stage COPD (EPIC): a phase II pilot RCT testing feasibility, acceptability, and effectiveness. J Pain Symptom Manage.

[18] Serious Illness Conversation Guide. - Ariadne Labs [Internet]. [cited 2023 Feb 9]. https://www.ariadnelabs.org/resources/downloads/serious-illness-conversation-guide/.

[19] Article Detail [Internet]. [cited 2023 Feb 9]. https://support.qsrinternational.com/nvivo/s/article/How-do-I-cite-QSR-software-in-my-work.

[20] Hsieh H-F, Shannon SE (2005). Three approaches to qualitative content analysis. Qual Health Res.

[21] Center to Advance Palliative Care. About palliative care [Internet]. [cited 2023 Jun 23]. https://www.capc.org/about/palliative-care/#:~:text=Palliative%20care%20is%20specialized%20medical,the%20patient%20and%20the%20family.

[22] Miravitlles M, Ribera A (2017). Understanding the impact of symptoms on the burden of COPD. Respir Res.

[23] Monteagudo M, Rodríguez-Blanco T, Llagostera M, Valero C, Bayona X, Ferrer M (2013). Factors associated with changes in quality of life of COPD patients: a prospective study in primary care. Respir Med.

[24] Dury R (2016). COPD and emotional distress: not always noticed and therefore untreated. Br J Community Nurs.

[25] Janssen DJ, de Hosson SM, bij de Vaate E, Mooren KJ, Baas AA (2015). Attitudes toward opioids for refractory dyspnea among Dutch chest physicians. Chron Respir Dis.

[26] Bernacki RE, Block SD, American College of Physicians High Value Care Task Force (2014). Communication about serious illness care goals: a review and synthesis of best practices. JAMA Intern Med.

[27] Brighton LJ, Bristowe K (2016). Communication in palliative care: talking about the end of life, before the end of life. Postgrad Med J.

[28] Maddocks M, Lovell N, Booth S, Man WD-C, Higginson IJ (2017). Palliative care and management of troublesome symptoms for people with chronic obstructive pulmonary disease. Lancet.

[29] Disler RT, Green A, Luckett T, Newton PJ, Inglis S, Currow DC (2014). Experience of advanced chronic obstructive pulmonary disease: metasynthesis of qualitative research. J Pain Symptom Manage.

[30] Curtis JR, Engelberg RA, Nielsen EL, Au DH, Patrick DL (2004). Patient-physician communication about end-of-life care for patients with severe COPD. Eur Respir J.

[31] Rising KL, Kemp M, Leader AE, Chang AM et al. A prioritized patient-centered research agenda to reduce disparities in telehealth uptake: results from a national consensus conference. Telemed Reports. 2023;4(1):387–395. 10.1089/tmr.2023.0051 PMC10758542 38169980

[32] Flieger SP, Chui K, Koch-Weser S (2020). Lack of awareness and common misconceptions about palliative care among adults: insights from a national survey. J Gen Intern Med.

[33] Iyer AS, Wells RD, Dionne-Odom JN, Bechthold AC, Armstrong M, Byun JY et al. Project EPIC (early palliative care in COPD): a formative and summative evaluation of the EPIC telehealth intervention. J Pain Symptom Manage. 2022. 10.1016/j.jpainsymman.2022.11.024 PMC10023469 36496113

[34] Vitacca M, Comini L, Tabaglio E, Platto B, Gazzi L (2019). Tele-assisted Palliative Homecare for Advanced Chronic Obstructive Pulmonary Disease: a feasibility study. J Palliat Med.

