# Supplementary material for: Perspectives from patients with chronic lung disease on a telehealth-facilitated integrated palliative care model: a qualitative content analysis study
Source: BMC Palliat Care. 2024 Apr 18;23:103. doi: 10.1186/s12904-024-01433-3 (PMC11027367; doi:10.1186/s12904-024-01433-3)
Supplement: Supplementary file 1 — Supplementary Material 1 [file 12904_2024_1433_MOESM1_ESM.docx]

**Appendix: Interview Guide**

Thank you for agreeing to participate in this research study. We would like to understand more about what people with chronic lung disease need to optimize their quality of life and plan ahead for if they get more sick. Sometime this includes palliative care.

What we mean by palliative care is: Palliative care is specialized medical care for people living with a serious illness. This type of care is focused on providing relief from the symptoms and stress of the illness. The goal is to improve quality of life for both the patient and the family. Palliative care is provided by a specially-trained team of doctors, nurses and other specialists who work together with a patient’s other doctors to provide an extra layer of support. Palliative care is based on the needs of the patient, not on the patient’s prognosis. It is appropriate at any age and at any stage in a serious illness, and it can be provided along with curative treatment.

We plan to use the information we collect to design a new model of care that uses telehealth to optimize the care that patients with chronic lung disease receive.

Do you have any questions or concerns before we begin?

I am going to turn the audio recorder on now... [TURN ON TAPE RECORDER]

THIS IS AN INTERVIEW WITH STUDY ID _____ BY INTERVIEWER ___________ ON ___________. [OPTIONAL] Also present is his/her caregiver ___________.

**GENERAL EXPERIENCE**

1. First let’s start with you telling me a little about yourself.
2. Next, I’d like to talk about your general experience with your lung disease. How does [DIAGNOSIS] affect your daily life?
3. What challenges and problems, have you faced in your daily life related to your [DIAGNOSIS]?

1. Tell me about the current care you receive from the Pulmonology team.
2. What problems do you wish your team could be more helpful for?
3. COVID-19 has increased the use of telehealth, defined as the delivery of health-related services via digital communication (e.g., video visits from your doctor on your computer, phone, or tablet). What is your experience with telehealth?
4. How comfortable are you with telehealth?
5. [IF PATIENT HAS BEEN RECENTLY HOSPITALIZED] I see that you were hospitalized in the past 6 months. Can you tell me more about what led to the hospital visit?
6. [IF PATIENT HAS BEEN RECENTLY HOSPITALIZED] Next, tell me about when you were discharged from (left) the hospital.
   1. Where did you go when you were discharged? (e.g. home, nursing home, skilled nursing)
   2. What challenges did you encounter after you left the hospital?
   3. What care/support did you have?
   4. What other care/support might have been helpful?

**KNOWLEDGE AND ATTITUDES**

Next, we are going to discuss palliative care. [Reiterate definition of palliative care]

1. Have you heard of palliative care before this?
   1. Have you ever received palliative care services either in the hospital or in the outpatient setting? Tell me about your experience with this.

***Here is a general definition that we use in healthcare to explain palliative care:*** Palliative care is specialized medical care for people living with a serious illness. This type of care is focused on providing relief from the symptoms and stress of the illness. The goal is to improve quality of life for both the patient and the family. Palliative care is provided by a specially-trained team of doctors, nurses and other specialists who work together with a patient’s other doctors to provide an extra layer of support. Palliative care is based on the needs of the patient, not on the patient’s prognosis. It is appropriate at any age and at any stage in a serious illness, and it can be provided along with curative treatment.

[**IF** patient asks if palliative care and hospice are the same]

Many people think palliative care and hospice are the same thing, but they are not. While hospice is a *type* of palliative care provided specifically at the end of life, palliative care can be provided to people of any age living with serious illness, and focuses on improving quality of life *at any stage of the disease process.* Palliative care may be called “supportive care” instead, and these terms are often used to mean the same thing. [***Check in***: Does this make sense?)

1. Do you think the addition of palliative care would be helpful to you for managing your symptoms and stress?
   1. Why or why not?

**EXISTING MODELS OF PALLIATIVE CARE**

There are several different models of where palliative care services are used: in your house, in the clinic, or in the hospital. These interactions focus on where you are with your disease and what are your goals for living with your disease and managing its impact on your quality of life, and then works with you and your pulmonary team to help make this happen.

**TIPC MODEL**

As I mentioned earlier, we plan to use the information we collect from you today to help develop a model of palliative care for people like you who are living with chronic lung diseases.

This model of palliative care that we are developing would work in the following way:

- **1)** First, you would have a video visit with a member of the XXX palliative care team and you would be able to talk about your illness and your needs related to this illness. This may include exploring your goals and preferences for your care, fears and worries, bothersome symptoms or limitations, and family involvement.
  - Questions here / Thoughts

- After this visit, **2)** the palliative care team would meet together to create a plan for how to adjust your care and share this with your pulmonary care team.
  - Questions here / Thoughts

- Finally, **3)** you would have video visit that includes you (and any family/caregiver that you want to include), the palliative care team and your pulmonary team. The goal of this visit will be to discuss the main goals that you identified in your initial visit, so that your pulmonary care team understands these goals, and possible changes in your treatment plan to better meet these goals.
  - Questions here / Thoughts

1. If you were able to have a 2-visit consultation with palliative care here at XXX, would you be interested in this service? Please explain your answer.
2. How could this model be made to better support your needs?
3. What things would make it difficult to receive this model of palliative care?
4. Is there anything we haven’t talked about that you think we should know about?

**I am turning off the recorder now, and I just have a few demographic questions to ask you.**
